# Could Testicular Tissue Be a New Arena for the Holmium Laser?

**DOI:** 10.7759/cureus.45234

**Published:** 2023-09-14

**Authors:** Mustafa Karaaslan, Mehmet Yilmaz, Melike Ordu, Mehmet Emin Sirin

**Affiliations:** 1 Urology, Bayındır Kavaklidere Hospital, Ankara, TUR; 2 Urology, Asklepios Klinik Triberg, Triberg, DEU; 3 Pathology, Faculty of Medicine, Aksaray University, Aksaray, TUR; 4 Urology, University of Health Sciences, Ankara Bilkent City Hospital, Ankara, TUR

**Keywords:** reproductive surgery, tunica layer, testicular tissue, laser effect, holmium:yag laser

## Abstract

Introduction

We aimed to observe the effects of holmium:yttrium-aluminum-garnet (Ho:YAG) laser on testicular tissue.

Methods

An ex vivo experiment was conducted using calf testicles. A 100 W laser generator with broad-spectrum settings of 10-80 W, 20-40 Hz, and 0.5-2 J, with a medium pulse duration, was tested. The laser effects on testicular tissues with and without the tunica layer were evaluated histopathologically by calculating the incision depth (ID), vaporization area (VA), coagulation area (CA), and total laser area (TLA=VA+CA) of the specimens.

Results

A total of 48 experiments were conducted. In testicular tissue without a tunica layer, the highest mean ID was determined at 1 J-20 Hz (0.247±0.0208 mm) and with a tunica layer at 2 J-40 Hz (2.673±0.032 mm). In the testicular tissue without a tunica layer, the highest mean VA was determined at 1.5 J-40 Hz (0.029±0.0016 mm^2^) and in tissue with a tunica at 2 J-40 Hz (6.173±0.114 mm^2^). The highest mean TLA in tissue without a tunica was detected at 2 J-20 Hz (0.038±0.0008 mm^2^) and in tissue with a tunica at 2 J-40 Hz (7.292±0.07 mm^2^). The mean ID, VA, CA, and TLA values of all the power outputs used were found to be statistically significantly higher in the testicular tissue with the tunica layer than in that without it (p<0.001).

Conclusion

The Ho:YAG laser has different effects on testicular tissue with and without a tunica layer. In testicular tissue without a tunica, the laser’s effect was minimal on the surrounding tissue, especially in terms of the ID, VA, and TLA. This minimal effect of the laser can be an advantage in testicular surgery procedures such as testis-sparing surgery (TSS) or testicular sperm extraction (TESE).

## Introduction

Laser technology is widely used in many areas of medicine for a variety of purposes [1-3]. In some cases, it has become an indispensable tool, especially in the field of endourology. Although various types of lasers are used in endourology, the holmium:yttrium-aluminum-garnet (Ho:YAG) laser has recently become the dominant instrument due to its effective and versatile structure [4,5]. The Ho:YAG laser, previously used only for stone surgery in urology, has been applied in many areas, from prostate surgery to tumor surgery, given its innovative features [6-8]. In recent years, the Ho:YAG laser has also been shown to be effective in soft tissues [6,7]. In endourology, courageous experiments continue to be carried out via ambitious efforts to open up new areas for this laser’s application and to consolidate its place in the field. However, laser technologies and their effects on tissue have largely been investigated in renal tissue in ex vivo studies [9-12].

Unlike other tissues among the urological organs, testicular tissue is highly vulnerable; it has been shown that testicular tissue and sperm production are sensitive to chemotherapeutic agents, radiotherapy, temperature, and even environmental pollution [13-16]. In some animal experiments, it has been reported that certain methods of sperm retrieval from the testicle may damage testicular tissue [17,18]. Although the impacts of these factors on testicular tissue have been studied, the effects of the Ho:YAG laser on this sensitive tissue are not yet known. In this ex vivo study, we aimed to observe the effects of the Ho:YAG laser on testicular tissue, thus exploring the limits of its use, aside from the urological tissues studied so far.

## Materials and methods

Ethics committee approval is not required for this study. The manuscript contains no clinical studies or patient data, nor does it contain any studies with human or animal subjects performed by any of the authors.

Experimental design and laser settings

**Figure 1:**
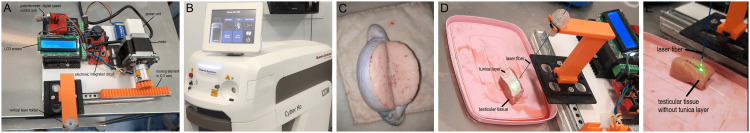
(A) A mini laser plotter robot system with laser holder, (B) 100 W Ho:YAG (Cyber Ho, Quanta System, Samarate, Italy) device, (C) cut calf testicle, and (D and E) experimental setup. LCD, liquid-crystal display; Ho:YAG, holmium:yttrium-aluminum-garnet

A mini laser plotter robot system that could move linearly and automatically along one axis (X-Y) was designed by MES (author) and Atıl Emre Cosgun (Figure 1A). The technical features, components, and working principle of the mini robot have been previously described in detail by our team [19].

A 100 W Ho:YAG laser device (Cyber Ho, Quanta System, Samarate, Italy) and 550 μm laser fiber were used for this study’s experiments (Figure 1B). According to the laser mode capacity, the following laser settings were selected: laser power in the range of 10-80 W, a frequency of 20-40 Hz, and an energy rate of 0.5-2 J. A medium pulse duration of 600 μs (50-1,100 μ) was used for the Ho:YAG applications. The descriptive features of the Ho:YAG laser and the settings applied during the experiments are presented in Table 1.

**Table 1:** The descriptive features of the Ho:YAG laser and the experimental setup details using mini laser plotter robot system Ho:YAG: holmium:yttrium-aluminum-garnet

Laser device	100 W Ho:YAG (Cyber Ho, Quanta System, Samarate, Italy)
Pulse modulation	Standard mode (SM)
Emission power (watts)	10-20-30-40-60-80
Pulse energy (J)	0.5-1-1.5-2
Frequency (Hz)	20-40
Pulse duration	Medium pulse, 600 μs each pulse
Laser fiber diameter (μm)	550
Automatic robotic arm speed	2 mm/second
Distance of fiber to the tissue	1 mm
Tissue fissure length	20 mm

In this ex vivo study, we used calf testes for laser application and histological examination. In the design of the study, the testicular tissue was prepared with and without a tunica layer, and laser application was performed on both tissues. The calf testes used in the study were obtained from freshly slaughtered calf cadavers at a slaughterhouse (Figure 1C). The testes were stored in 0.9% saline at 2°C-6°C without peeling the tunica layer until the experiments were started, and the laser was turned on. For each experiment, the calf testes were cut into 3×2×2 cm blocks. In some of the cut tissues, the tunica layer was peeled off with the help of a scalpel. Testicular tissues with and without a tunica layer (i.e., inner testicular layer) were placed on a plastic board fixed on the table before laser application (Figure 1D, 1E). The speed of the X-Y axis of the mini robot was set to 2 mm/second. The laser fiber was placed in the laser holder and moved in the X-Y direction at a speed of 2 mm/second, 1 mm away from the testis tissue. A 20 mm incision was then made on the testicular tissue. Before each experiment, the laser fiber was renewed by cutting it with ceramic scissors.

Histopathological measurements after laser application

**Figure 2:**
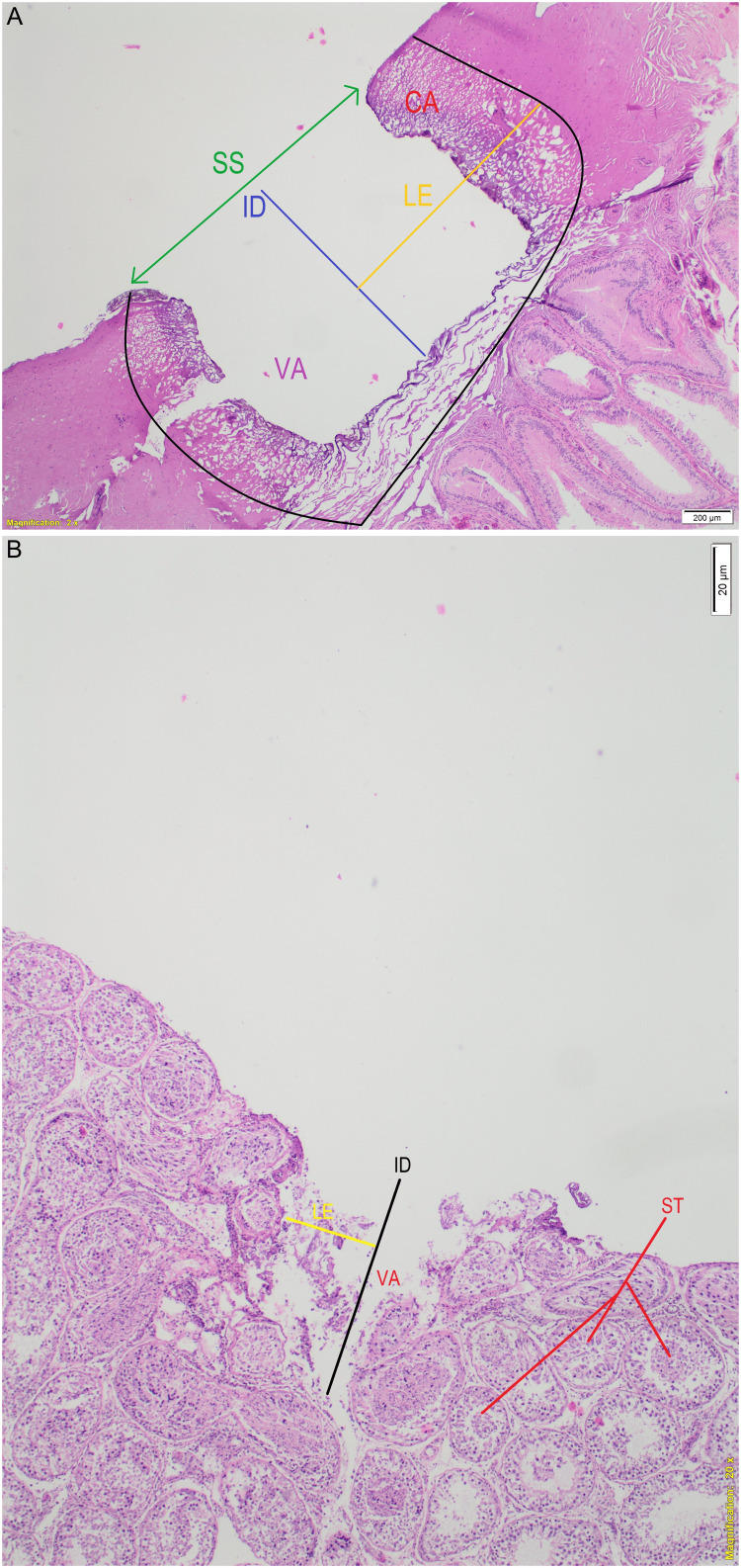
(A) Histopathological measurements of Ho:YAG on testicular tissue with tunica layer: lateral effect (LE), surface section (SS) (which is defined as a thin horizontal section of the superficial part of the tissue affected by the laser), incision depth (ID), vaporization area (VA), coagulation area (CA), and total laser area (TLA=VA+CA). (B) Histopathological measurements of Ho:YAG on testicular tissue without tunica layer (i.e., inner testicular layer). The laser setting used in the images is 1.5 J-40 Hz. ST, seminiferous tubules; Ho:YAG, holmium:yttrium-aluminum-garnet

After each experiment, the testes were preserved in 10% buffered formaldehyde. The laser fissures of each specimen were cut vertically and horizontally, and both parts were embedded in paraffin blocks. The tissue blocks were cut into five 50 µm rounds, mounted on glass, and stained with hematoxylin and eosin. The width, depth, and area of laser penetration into the tissue were blindly assessed by an experienced pathologist (MO) using an electronic light microscope (Olympus BX53-SC100, Olympus Europa SE & Co. KG, Hamburg, Germany). Incision depth (ID), vaporization area (VA), coagulation area (CA), total laser area (TLA=VA+CA), surface section (SS), and lateral effect (LE) were measured blindly by the same pathologist using the cellSens imaging software (Olympus Europa SE & Co. KG, Hamburg, Germany) (Figure 2). SS was defined as a thin horizontal section of the superficial part of the tissue affected by the laser, and LE was the longest horizontal radius from the center of the vaporization gap to normal tissue, including the coagulation zone. The results of the histopathological measurements obtained from the laser applications were then compared.

The Statistical Package for Social Sciences (SPSS) version 22.0 (IBM SPSS Statistics, Armonk, NY) was used to statistically analyze the research data. The data are presented as means±standard deviations (SDs).

## Results

**Figure 3:**
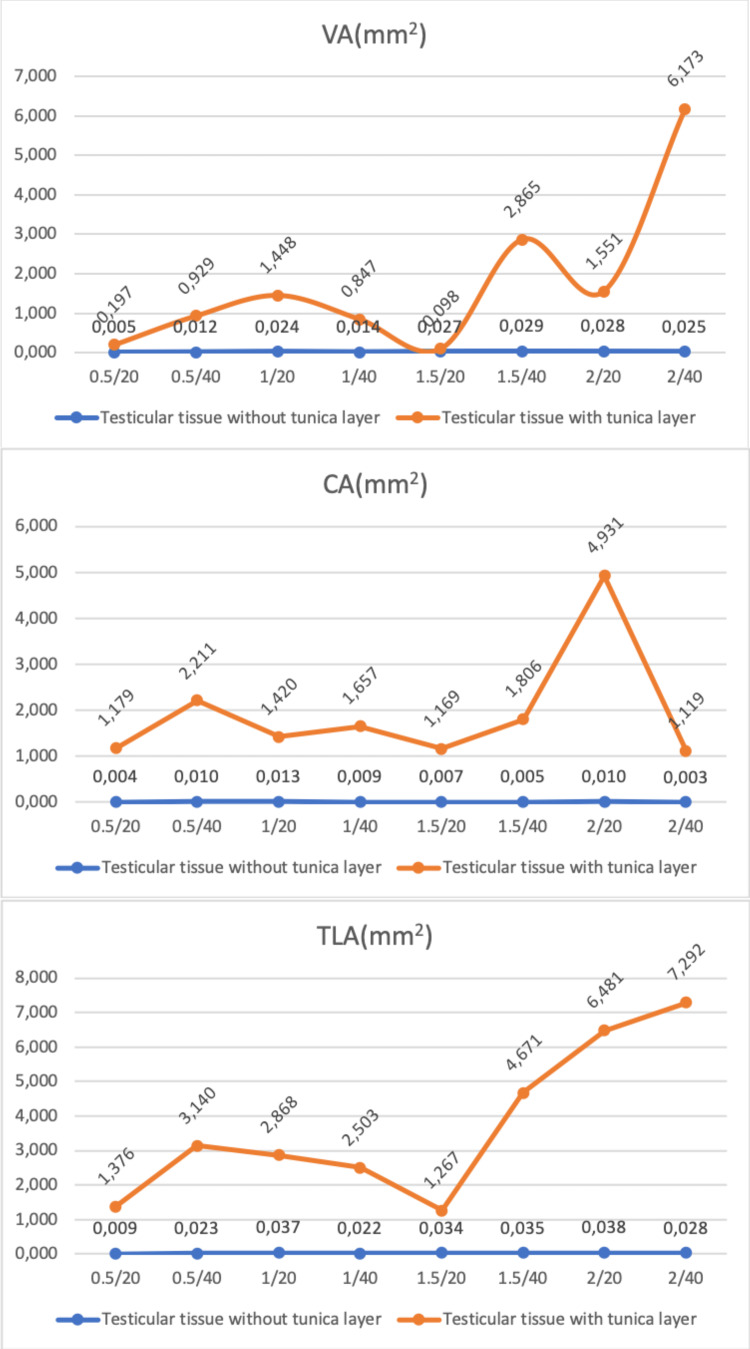
Vaporization area (VA), coagulation area (CA), and total laser area (TLA=VA+CA) values of different laser settings of Ho:YAG on testicular tissue with and without tunica layer. Ho:YAG: holmium:yttrium-aluminum-garnet

**Figure 4:**
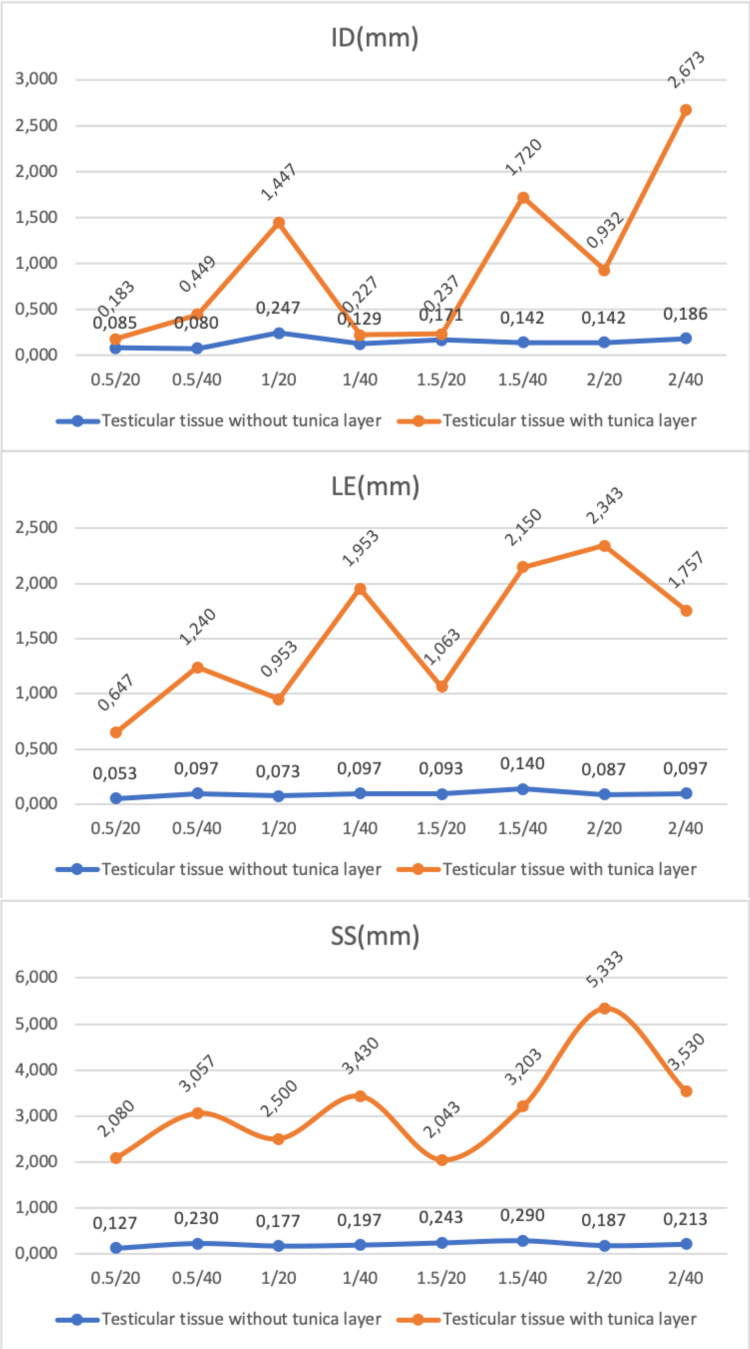
Incision depth (ID), lateral effect (LE), and surface section (SS) values for various laser settings of Ho:YAG on testicular tissue with and without tunica layer. Ho:YAG: holmium:yttrium-aluminum-garnet

In the study, each experimental set was replicated three times, and a total of 48 experiments were conducted. The mean histopathological measurements (VA, CA, TLA, ID, LE, and SS) of the Ho:YAG laser application on testicular tissue with and without a tunica layer according to the laser settings used in the air environment are presented in Figure 3 and Figure 4.

In the testicular tissue without a tunica layer, the highest and lowest mean VAs were determined at the laser settings of 1.5 J-40 Hz and 0.5 J-20 Hz (0.029±0.0016 mm^2^ and 0.005±0.0004 mm^2^, respectively). In testicular tissue with a tunica layer, the highest and lowest mean VAs were determined at the laser settings of 2 J-40 Hz and 1.5 J-20 Hz (6.173±0.114 mm^2^ and 0.098±0.002 mm^2^, respectively). The mean VA value of all power outputs used was found to be statistically significantly higher in the testicular tissue with the tunica layer than in that without it (p<0.001). In testicular tissue without the tunica layer, the highest and lowest mean CAs were determined at the laser settings of 1 J-20 Hz and 2 J-40 Hz (0.013±0.0022 mm^2^ and 0.005±0.0005 mm^2^, respectively). In testicular tissue with a tunica layer, the highest and lowest mean CAs were determined at the laser settings of 2 J-20 Hz and 2 J-40 Hz (4.931±0.056 mm^2^ and 1.119±0.085 mm^2^, respectively). The mean CA value of all power outputs used was found to be statistically significantly higher in the testicular tissue with the tunica layer than in the testicular tissue without the tunica layer (p<0.001). In tissue without the tunica layer, the highest and lowest mean TLAs were recorded at the laser settings of 2 J-20 Hz and 0.5 J-20 Hz (0.038±0.0008 mm^2^ and 0.009±0.001 mm^2^, respectively). In the tissue with a tunica layer, the highest and lowest mean TLAs were detected at the laser settings of 2 J-40 Hz and 1.5 J-20 Hz (7.292±0.07 mm^2^ and 1.267±0.012 mm^2^, respectively). The mean TLA value of all power outputs used was found to be statistically significantly higher in the testicular tissue with the tunica layer than in that without it (p<0.001).

The highest and lowest mean IDs for tissue without the tunica layer were determined at the laser settings of 1 J-20 Hz and 0.5 J-40 Hz (0.247±0.0208 mm^2^ and 0.08±0.003 mm^2^, respectively). In testicular tissue with a tunica layer, the highest and lowest mean IDs were recorded at the laser settings of 2 J-40 Hz and 0.5 J-20 Hz (2.673±0.032 mm and 0.183±0.005 mm, respectively). The mean ID value of all power outputs used was found to be statistically significantly higher in the testicular tissue with the tunica layer than in that without it (p<0.001). The highest and lowest mean LEs were determined at the laser settings of 1.5 J-40 Hz and 0.5 J-20 Hz (0.14±0.01 mm and 0.053±0.0031 mm, respectively) for tissue without the tunica layer. In tissue with a tunica layer, the highest and lowest mean LEs were recorded at the laser settings of 2 J-20 Hz and 0.5 J-20 Hz (2.343±0.031 mm and 0.647±0.031 mm, respectively). Again, the mean LE value of all power outputs used was determined to be statistically significantly higher in the tissue with the tunica layer than in that without it (p<0.001). Finally, the highest and lowest mean SSs were determined at the laser settings of 1.5 J-40 Hz and 0.5 J-20 Hz (0.290±0.009 mm and 0.127±0.0043 mm, respectively) for tissue without the layer. In tissue with the layer, the highest and lowest mean SSs were recorded at the laser settings of 2 J-20 Hz and 1.5 J-20 Hz (5.333±0.137 mm and 2.043±0.041 mm, respectively). The mean SS value of all power outputs used was statistically significantly higher in the testicular tissue with the tunica layer than in that without it (p<0.001).

## Discussion

In the present study, we demonstrated the histopathological effects of the Ho:YAG laser on calf testis tissue through an ex vivo experimental design. To the best of our knowledge, ours is the first experimental study in the literature to examine the Ho:YAG laser effects on testicular tissue.

In our study, we examined the effects of the laser on two types of testicular tissue: with and without the tunica layer (i.e., inner testicular layer). We believed that by comparing the testicular tissues with and without the tunica, we would be able to more clearly observe the effectiveness of the laser in the tissue without the tunica, which was important in determining its direct effect on the testicular tissue. We found that ID was much smaller in testicular tissue without the tunica layer. For all laser settings, the ID in the tissue without the tunica was found to be less than 0.2 mm. Considering that ID represents the cutting property of the laser, the incision depth of the Ho:YAG laser applied directly to the testicular tissue (i.e., without the tunica layer) was quite short. This was probably due to the fact that the seminiferous tubules contain a relatively large amount of fluid, which absorbs a large part of the laser’s energy, because laser beams reaching the tissue are absorbed by hemoglobin, water, and melanin. In tissues characterized by a high tissue absorption coefficient, such as those with high fluid content, the laser beam exhibits shallow penetration, whereas in tissues with a low tissue absorption coefficient, it exerts a deeper effect [20].

In addition, the fact that TLA is quite low in testicular tissue without a tunica layer can be attributed to the low level of damage caused by the laser around the target tissue. Regarding testicular tissue with a tunica, we found that ID increased at settings where the energy was constant at 0.5 J, 1.5 J, and 2 J and the frequency increased. This may be explained by the fact that thermal relaxation and thus the cooling effect are lower at high frequencies [21]. In other words, low thermal relaxation leads to an increase in the effect of the laser on the tissue.

It should be noted that the same power (W) value did not have the same effect on the tissue types [9]. In tissue with a tunica, we observed that ID increased with increasing frequency at energy values of 1.5 J and 2 J. However, the same observation was not valid for testicular tissue without a tunica. In general, the ID effect of the laser was more pronounced in tissue with a tunica than that without it, and the increases between laser settings were relatively more dramatic in the case of the former. At high wattages (40, 60, and 80 W), LE and SS increased as ID decreased, and LE and SS decreased as ID increased in tissue with a tunica layer. Since the amount of fluid contained in the tunica layer is relatively low, there is little fluid to absorb the energy, so the energy shows its effect either on the surface or on the sides.

In our study, we observed that not all W values had the same effect on VA. For example, there was approximately a twofold difference in VA between 0.5 J-40 Hz and 1 J-20 Hz in both tissue types. Moreover, there was not always a linear relationship between W and VA. At 1 J-20 Hz, VA was 0.024 mm^2^ in tissue without a tunica and 1.448 mm^2^ in tissue with a tunica, and these values were reduced to half at 1 J-40 Hz (although W was doubled). At the 1 J energy setting, this can be explained by the cooling effect [21]. Similarly, we found that the effect of increasing frequency on the tissue varied according to the J value. At 2 J energy, as the frequency increased (from 20 Hz to 40 Hz), we found that VA increased in the tissue with a tunica. In the tissue without it, VA values did not differ much at energies of 1.5 J and 2 J. These observations were also valid for TLA. Therefore, we would like to emphasize that the J values should be at certain thresholds to change the effect of the frequency increase on the tissue. Interestingly, at a laser setting of 1.5 J-20 Hz, significant decreases in ID, VA, CA, and TLA values were observed. This can be attributed to the multiple structural features of the tunica layer and the fact that it is not always of the same thickness. The tunica structures in the testis consist of mesothelial cells (tunica vaginalis), collagen fibers, myocytes, fibroblasts (tunica albuginea), and connective tissue consisting of vessels and lymphatics (tunica vasculosa) [22]. In our study, the tunica thickness ranged from 3.5 to 11 mm. Additionally, the effects of high wattage on ID, LE, and SS were clearly seen in testis tissue with a tunica layer.

It is worth briefly reviewing how this ex vivo study will be reflected in some clinical applications. In this regard, one may ask why we need to see the effects of a laser, even though a scalpel and cautery are available for testicular surgical procedures, such as testis-sparing surgery (TSS) or testicular sperm extraction (TESE). In the European Association of Urology (EAU) 2023 guidelines, it is strongly recommended to discuss TSS with frozen section examination in patients with a high probability of benign testicular tumors suitable for enucleation [23]. With a better knowledge of the effects of lasers on testicular tissue, the patient profile in which TSS can be applied may expand, and it may therefore be recommended to a wider patient group, not only in those who are suitable for enucleation. With regard to TESE, the fact that the laser also has a coagulation effect and that it causes less damage to the surrounding tissue with low TLA values during application may be advantages for its use in this procedure.

Study limitations

Our study has some limitations that should be addressed. First, the testicular tissues to which we applied the laser in the experiments were tissues without perfusion. This restricts the ability to draw conclusions regarding the coagulation, ablation, heat absorption, and vaporization effects of the laser on the blood-supplying tissue. Therefore, our results should be evaluated in an ex vivo context. Second, since there may be variations in the amount of semen (and therefore fluid content) in the seminiferous tubules in the cut testicular tissue blocks, there may have been differences in the histopathological parameters between laser-applied testicular tissue blocks with the tunica layer. Third, the effects of laser application on spermatozoa were not evaluated in the present study. Finally, due to the technical features of the laser device, it was necessary to keep the laser settings in the experiments within the specified ranges.

## Conclusions

Despite the study’s limitations, we were able to demonstrate the effect of the laser on testicular tissue. In testicular tissue without a tunica layer, the effect of the laser on the tissue was acceptable, with minimal effect on the surrounding tissue, especially as indicated by the ID, VA, and TLA. This can be interpreted as a relatively harmless range of effects of the Ho:YAG laser on testicular tissue without a tunica layer. Furthermore, our study also provides an outline of the limits of the use of the Ho:YAG laser in urology. Undoubtedly, as the number of studies on the effects of the Ho:YAG laser on testicular tissue increases, we will be able to obtain more precise data about the effects of the laser on testicular tissue. In this regard, we believe that our study provides a basis for future studies on the effects and use of the Ho:YAG laser on testicular tissue.
